# American Dental Association

**Published:** 1897-03

**Authors:** 


### Reports of Society Meetings.

### AMERICAN DENTAL ASSOCIATION.

(Continued from page 119.)

*August 6, 1896.—Third Day.—Afternoon Session.*

THE meeting was called to order at five o'clock, the President, Dr. John Y. Crawford, occupying the chair.

The minutes of the previous session were read and approved.

*Dr. Peirce.*—It is the duty of the Executive Committee to make a report regarding places for meeting next year. This duty devolves upon the section of which I am chairman. I hold in my hand papers sent here from Denver, Colorado, inviting this Association to meet there next year. I have first to present to you a petition signed by seventy dentists, offering every opportunity that the profession could ask for holding a successful convention. I have also a petition from the hotels, offering rooms and the best of service that can be had at from three to five dollars per day. I have a lengthy communication from the Chamber of Commerce, begging us to accept the courtesies of the city, and offering every possible opportunity for comfort and success; also from the mayor of the city and from the governor of the State. The business men have also sent a petition, and the railroads say they think they can carry people from the East to Denver for about sixty dollars, the price of one fare. I am told that the nights there are always cool, and that it could not possibly be as hot as here. The mountains are close by, and any one finding the city too warm can go to the mountains.

I am also asked by the committee to request you to meet at Old Point Comfort. The Southern Dental Association have selected Old Point Comfort, and they desire us to meet at the same time, hoping some combination can be made.

Washington and Niagara Falls have also been mentioned, but I trust some one interested in the union of the societies will take the floor and state the advantages of meeting at Old Point Comfort.

*Dr. Fillebrown.*—Two years ago this committee was appointed to invite the Southern Dental Association to appoint a similar committee to confer in regard to the combination of the two societies. Last October the Southern Dental Association responded to our invitation and appointed a committee of five members. Aside from that, at the last annual meeting held at Asheville, North Carolina, on July 28, they voted to continue that committee and to further consider the matter, and to report finally at a subsequent meeting, and they also voted to meet at Old Point Comfort, with the idea that it would be convenient for the American Dental Association to meet there with them, and expressed formally the desire to have us meet there. We were the hosts in the beginning, and invited them, through the action of the committee, to do what they did. They have fixed Old Point Comfort, and they would have invited us to meet there, but the idea was expressed that that Association had no especial right to invite this Association to meet on the soil of Virginia when they were an Association that was meeting in Asheville, North Carolina. They want us to consider the subject, and every man, I think, hopes it may be brought about. The Hygeia Hotel has placed in the hall we occupy a system of electric fans, which makes the place comfortably cool. There is also a new hotel which is very fine in its appointments, and there is an old hotel which has been renovated, the charges to be two dollars a day. At the Hygeia it will be about two dollars and fifty cents. Four dollars a day is the regular rate at the Chamberlain, but they would do a little better for us.

*Dr. Morrison.*—I would like to ask if the committee has not discretionary power as to meeting either at Old Point Comfort or any place that may be selected by the Association. My understanding was that there was some discretionary power granted to call the Southern Dental Association to meet wherever we meet next year.

*Dr. Richards.*—The action of the society, as I understand it, was that they were to meet substantially as set before you by Dr. Fillebrown, hoping that this Association would meet them at that point. Our society is of course subject to the action of its Executive Committee. The chairman, I believe, is present,—Dr. Clifton, of Texas. I do not remember any motion to the effect of changing from that point to any other. We, unfortunately, in the past year or two, have been going about somewhat, and it seems to me, as far as I remember, that no provision was made for any other place, thinking that Old Point Comfort would suit all parties better than any other we might select.

*Dr. Moore.*—Before coming here I had not learned of the meeting of the Southern Dental Association at Old Point Comfort. Knowing the matter of the union of the two societies had been talked over by the committee, and it was hoped it might be consummated next year, after consultation with a number of gentlemen from different sections of the country, I wrote Mr. Pike, the proprietor of the Hygeia Hotel, asking him if he could accommodate us next year, and at what rate he would give us board, and if he could give us all the accommodations we would need in the way of rooms for meeting purposes, committee-rooms, etc., for the Association of Faculties and the Board of Examiners, and asked him if he wanted us to come to telegraph me. He answered yesterday, and said he would make the necessary arrangements, and give us accommodations for two dollars and fifty cents per day. His regular rates are higher than that. Some of the gentlemen proposed that I should telegraph to the proprietor of the Chamberlain, which is one of the finest hotels in the South. Their regular rates are four dollars per day. I have authority from the Virginia State Society to invite this Association to come to Old Point Comfort.

*Dr. Morrison.*—I want to second the nomination of Denver as the place of meeting, from the fact that it is a little east of the geographical centre of this country, and the Eastern division of the country is having a little more of the benefits of the Association than any other portion of the country. The West is entitled to a little more representation, and I can assure you, from my knowledge of the people of Denver, that you will be well entertained, and it will be a revelation for the Eastern gentlemen to pay a visit to that beautiful city at the foot of the Rockies.

*Dr. Rhein.*—I want to nominate a place for the next meeting of this Association, and in doing so I would say, if I thought there was any possibility of a successful consolidation of this Association with the Southern Dental Association, I would gladly support the movement to meet at Old Point Comfort; but I do not believe, from all I have learned, that the majority of the Southern members are willing to give up their Association. Their remarks in that direction at Atlanta show that the majority of them felt that way. We all have had, I believe, by mail, an invitation to hold our next meeting in California, and I am thoroughly in favor of taking the next meeting there. I see no reason why we should stop at Denver. It is about time we went West. We have talked about it for years. Therefore I nominate the city of San Francisco for the next meeting.

*Dr. Mason.*—I think if the gentleman had ever ridden from Denver to San Francisco in the train he would not care to take the ride again very soon.

*Dr. Molyneaux.*—I second the nomination made to go to Old Point Comfort for the very reason that we never know what we can accomplish until we try. It seems to me if there is a sentiment of antagonism to this idea of joining forces with the Southern Dental Association, if we meet these people half-way, they will change their feeling, and we will unite in one grand society. Therefore while we have been there but recently, I feel like seconding the nomination that we go there again, and see if we cannot further this matter.

*Dr. Davis*, of Chicago.—I rise to second the nomination for Denver, for exactly the same reasons that the gentleman has already seconded the nomination for Old Point Comfort. When we met in Minnesota our membership was five hundred or more, and now it is only about two hundred. It has been shown from the two meetings held in the West that the West is the place to get the membership. If we do not meet West, the chances are that we will have a Western Dental Association, and I think in seconding the nomination for Denver that the Californians will feel that they have an interest and a chance to meet with us. It is as near the centre of the two points as it can be, and is central for all parties. If the Southern Dental Association want to affiliate with the American Dental Association, I see no reason why they should not meet in Denver.

*Dr. Templeton.*—I move that the nominations close.

Drs. McKellops and Molyneaux were appointed as tellers.

Sixty-nine votes were cast, of which Old Point Comfort received 45; Denver, 17; San Francisco, 1; Niagara Falls 5; Norfolk, 1.

ELECTION OF OFFICERS.

Dr. Cushing moved that the first ballot be an informal or nominating ballot. If upon the second ballot there should be no choice of names, the highest three shall be taken, and at each subsequent ballot the name having the smallest number of votes shall be dropped, this rule to apply to all the nominations.

Motion carried.

The balloting resulted as follows:

*For President.*—Fifty-seven votes were cast, of which Dr. Truman received 32; Dr. Fillebrown, 17; Dr. Watkins, 2; Dr. McManus, 3; Dr. Hunt, 1; Dr. Clifton, 1; Dr. Templeton, 1.

A motion was made to make this ballot a formal one.

Motion carried.

*For Vice-President.*—Fifty votes were cast, of which Dr. Fillebrown received 40; Dr. Watkins, 3; Dr. Clifton, 4; Dr. H. A. Smith, 1; Dr. Morrison, 1; Dr. D. Holly Smith, 1.

A motion was made to declare the ballot a formal one.

Motion carried.

*Second Vice-President.*—Forty-four votes were cast, of which Dr. Clifton, of Waco, Texas, received 33; Dr. Watkins, 1; Dr. Morrison, 8; Dr. Hunt, 1; Dr. Richards, 1.

Motion made to make the ballot a formal one.

Motion carried.

Dr. Peirce moved that the President cast one vote for Dr. Cushing for Recording Secretary.

Motion carried.

The same motion was made in reference to the Corresponding Secretary, and Dr. Emma Eames Chase was re-elected.

The Treasurer, Dr. Morgan, of Nashville, Tennessee, was also re-elected in the same manner.

The next order of business was the election of three members of the Executive Committee in place of Drs. W. W. Walker, D. N. McElhenny, and S. G. Perry.

Upon a ballot being taken, Dr. Perry received 38 votes; Walker, 26; and A. O. Hunt, 29.

A motion was made to make the ballot a formal one.

Motion carried.

*Dr. Boice.*—I have a resolution that I would like to offer:

"Resolved, That our Recording Secretary have printed a list of members, with their addresses; also a list of the members of the sections, and send the same to each member on or before November 1, 1896."

Resolution adopted.

The chairman of Section I. requested the privilege of drawing upon the Treasurer for one hundred dollars, as he had some plans for the improvement of the section. The matter was referred to the Executive Committee.

A vote of thanks was offered to the proprietor of the Grand Union Hotel for the courtesies shown to the Association.

Drs. Richards and Gaylord were appointed to conduct the newly-elected President to the chair.

*Dr. Crawford.*—Permit me to return to you my sincere thanks for the uniform courtesy and confidence you have extended to me during the last two years of your history. It now affords me pleasure to welcome to this platform a gentleman so distinguished in the learning of our profession, and not only in that, but all of its collateral sciences,—not only distinguished in these departments, but also as a teacher and an educator, and one whom I have no doubt will preside over this society with much honor.

*Dr. Truman.*—Permit me to thank you most sincerely for this high honor, entirely unexpected when I entered this hall in the earlier portion of this week. No idea of being President of this Association entered my mind. I am satisfied that it was done without any political influence, and as I hold and have ever held that the place must seek the man, and not the man the place, I accept it with satisfaction, and, as I said before, with thanks.

It seems to me that there is something to be done by this American Dental Association to overcome the apathy which has hovered like a cloud over it for several years past. We all love this body; we want to see it energetic and moving in proper lines towards higher things; and the only way to accomplish that is through enthusiastic efforts. It seemed to me last year, when we met at Asbury Park, that the system was defective which permitted a large number of papers to be read by title. It is always discouraging to a man to have his paper disposed of in this way. What has been the result? We have had a paucity of papers this year, and while, to me, the forepart of this meeting was the most satisfactory of any I have attended, I cannot speak so of the latter part, because both mechanical and operative branches were almost entirely ignored. We have many men of different minds in our Association, and we must cater to all shades of thought. Therefore I hope that during the next year, and in the early part of the year, there will be an effort made to bring out all the sections in full force. For myself, I cannot be at the head of anything that seems to be in a dying condition. I know by proper energy this Association can be brought up to full fruition.

I do not wish to detain you to-day. It is growing late, and I am satisfied when we come to meet at Old Point Comfort the hope of many years of my later life will be accomplished, and we will join hands with our Southern brethren and come up as one body to carry on this great work in the future.

The chair appointed as a local committee for the ensuing year, Drs. J. Hall Moore and T. H. Parramore.

*Dr. Crawford.*—I would like to say a word. We have done the proper thing in re-electing our Corresponding Secretary. I want to call the Executive Committee's attention to her efficiency in this work. She used her influence in corresponding with the local societies, in providing blanks at the proper time, in advance of the meeting of the State societies, in order that the applications might come in in due form. I want to say this word of commendation for her willingness and her efficiency to do that work.

The chair appointed Drs. E. T. Darby and A. W. Harlan on the Publication Committee.

The final reading of the minutes then took place, after which the Association adjourned to meet at Old Point Comfort on the first Tuesday of August, 1897.

